# Assessing geographical distribution and accessibility of emergency obstetric care in sub-Saharan Africa: a systematic review

**DOI:** 10.7189/jogh.09.010414

**Published:** 2019-06

**Authors:** Aduragbemi Banke-Thomas, Kikelomo Wright, Lindsey Collins

**Affiliations:** 1 Centre for Reproductive Health Research and Innovation, Lagos State University College of Medicine, Ikeja, Lagos, Nigeria; 2 Department of Health Policy, London School of Economics and Political Science, London, UK; 3 McCain Institute for International Leadership, Arizona State University, Tempe, Arizona, USA; 4 Department of Community Health and Primary Health Care, Lagos State University College of Medicine, Ikeja, Lagos, Nigeria; 5 Decision Theatre Network, Arizona State University, Tempe, Arizona, USA; 6 School of Geographical Sciences and Urban Planning, Arizona State University, Arizona, USA; **Background** In sub-Saharan Africa (SSA), over 200 000 women (66% of global figures) die annually due to complications of pregnancy and childbirth. Many of these deaths are preventable, especially if women have access to timely emergency obstetric care (EmOC). With poor roads and difficult topography in the region, access can be impeded. Based on United Nations EmOC assessment guidelines, minimum acceptable levels for geographical distribution of EmOC facilities have been defined (EmOC Indicator 2). We aimed to critically assess studies published in the peer-review literature that assessed EmOC geographical distribution and accessibility in SSA.

## Abstract

**Methods:**

Two reviewers systematically searched multiple databases for articles published between January 2009 and June 2018. Both screened and selected studies based on the set inclusion criteria. Following quality assessments, data on study characteristics, process of data collection and analysis and findings reported were extracted. Extracted data were synthesised and presented in tables and charts. Narrative synthesis was used to summarise reported findings.

**Results:**

15 studies met the inclusion criteria, with varying assessed quality: high (7 studies), medium (4 studies) and low (4 studies). 8 studies were conducted at a national level while 7 were sub-national. 8 studies focused on assessing Indicator 2, while the others assessed multiple EmOC indicators. Only about half of the studies presented details of analysis for assessing geographical distribution, provided a map and interpreted their findings. Similarly, half of the studies used geographic information systems (GIS) for analyses. Of these, GIS was used to map EmOC facilities or relate facility numbers to 500 000 population (3), estimate straight-line distances between facilities and residences of women (2) and model travel scenarios (3). EmOC facilities in SSA are concentrated in capitals, central and urban areas and at least a third of women in the region cannot reach their nearest EmOC facility within the recommended two-hour time-frame.

**Conclusions:**

There is a limited number of studies that have assessed EmOC geographical distribution in SSA. When available, completeness and quality of analysis are questionable. Comprehensive assessments need to maximise recent advancements in mobile and GIS open-source technology to provide more realistic representation of EmOC access for service planners and policy-makers.

**PROSPERO Registration:**

CRD42018099882

Despite global efforts aimed at maternal mortality reduction, 303 000 women still lose their lives due to complications of pregnancy and childbirth annually. Ninety-nine percent of these deaths occur in low- and middle-income countries (LMICs), with the sub-Saharan African region accounting for almost two-thirds of the recorded maternal deaths [1]. Similar to other regions, direct obstetric complications which usually present as emergencies such as haemorrhage, hypertension, sepsis, complications of obstructed labour, and unsafe abortion lead to more than three-quarters of these deaths in sub-Saharan Africa (SSA) [2]. It has long been established that three delays are associated with maternal deaths: delay in decision to seek care, delay in travel to an appropriate health facility, and delay in receiving appropriate care upon arrival at the facility [3]. When and if women arrive at health facilities, evidence suggests that provision of timely and quality emergency obstetric care (EmOC) significantly reduces maternal morbidity and mortality, that could otherwise occur [4]. EmOC can either be basic (comprising of seven care packages – injectable antibiotics, injectable oxytocics, injectable anticonvulsants, manual removal of placenta, removal of retained products, assisted vaginal delivery and basic neonatal resuscitation), or comprehensive – all basic EmOC packages plus blood transfusion and caesarean section) (**Table 1**) [5].

**Table 1:** Emergency obstetric care (EmOC) signal functions*

Signal functions	
**Basic emergency obstetric care (BEmOC)**	**Comprehensive emergency obstetric care (CEmOC)**
1) Antibiotics (injectable)	All Basic EmOC functions (1-7) plus
2) Oxytocics (injectable)	8) Caesarean
3) Anticonvulsants (injectable)	9) Blood transfusion
4) Manual removal of placenta	
5) Removal of retained products	
6) Assisted vaginal delivery	
7) Basic neonatal resuscitation	

*A BEmOC facility is one in which all functions 1-7 are performed. A CEmOC facility is one in which all functions 1-9 are performed.

In 2009, the World Health Organization (WHO) updated the guidelines for assessing the availability and quality of EmOC, recommending eight indicators, one of which included ‘geographical distribution of EmOC facilities’ (EmOC Indicator 2) [5]. The WHO recommended that as an acceptable minimum level for geographic distribution, “all subnational areas have at least five emergency obstetric care facilities (including at least one comprehensive facility) for every 500,000 population” [5]. In the guideline, the WHO also recommended that to assess the indicator, researchers should “calculate the distribution of EmOC facilities for subareas” and report the percentage of the subareas meeting the acceptable minimum levels. In addition, researchers could map the facilities in sub-areas and show roads as well as the general topography, using geographical information system (GIS) or similar mapping methods. This may be more useful for health system planners to “identify problems of access and show referral systems” [5]. While such additional analysis was recommended, no specific indicator was proposed to assess it [5].

For varied reasons in SSA urban and rural areas, geographical distribution and accessibility of EmOC facilities is particularly critical. In many urban areas in the region, there are high population densities mostly due to urbanization [6]. Additionally, roads, which make up the dominant mode of motorized transport in SSA, are mostly in poor conditions and therefore prone to severe traffic congestions [7]. In the rural areas, roads are in even poorer conditions, compounded by difficult terrain [8]. These factors limit access to EmOC facilities if women decide to seek care and may jeopardise pregnancy outcomes [9,10]. With the persistent high maternal mortality burden in SSA, it is critical to understand how geographical availability and access to EmOC facilities have been measured. Our objective in this systematic review was to explore studies published in the peer-review literature that assessed EmOC geographical distribution and accessibility in SSA.

## METHODS

### Search strategy

Using the Preferred Reporting Items for Systematic Reviews and Meta-Analyses (PRISMA) approach [11], PubMed, Scopus, Embase, CINAHL, Global Health and Directory of Open Access Journal (DOAJ) were searched for articles published between year 2009 (the publication year of the updated WHO handbook [5]) and June 2018 (The PRISMA Checklist is provided in Appendix S1 in **Online Supplementary Document**). The search terms used combined the care package (“Emergency Obstetric Care” OR “Emergency Obstetric and Newborn Care” OR EmOC OR EmONC) AND a word indicating assessment of the care package (Assess* OR evaluat* OR monitor* OR function* OR perform* OR effect*). Duplicates were identified and removed. Subsequently, a reference-list checking of the retrieved articles was conducted to identify any additional relevant articles that had been missed. The search was conducted by two authors (ABT and LC). Following title and abstract screening to confirm relevance of articles, ABT and LC independently read the articles to identify those that specifically assessed EmOC geographical distribution and accessibility in sub-Saharan African countries, as defined by the World Bank [12]. Both authors compared their retrieved records and agreed on final eligibility based on the agreed inclusion/exclusion criteria. Any disagreements were resolved through discussions with another author (KW).

### Inclusion and exclusion criteria

Articles were included if they reported observational studies that assessed EmOC geographical distribution and accessibility and were published in the peer-reviewed literature from January 2009 to June 2018. Articles had to be published in English language and conducted in a sub-Saharan African country to be included. Commentaries, editorial letters, conference proceedings, other reviews and studies that solely focused on testing associations between travel/access to specific facility or facilities and dependent variables, were excluded.

### Data extraction and synthesis

Articles that met the inclusion criteria were allocated unique identifiers for auditing. Article full-texts were subsequently reviewed, and data extracted using a pre-developed extraction sheet. Data collected included the author(s), publication year, study country, stated study objectives, scale of study (national, sub-national or facility level), indicators assessed, number of facilities included, percentage of all facilities surveyed, level of care (Basic Emergency Obstetric Care (BEmOC) or Comprehensive Emergency Obstetric Care (CEmOC)), type of facilities included (public or private), assessment model used (United Nations (UN) EmOC assessment model or others), data sources, methods used for collecting data to assess EmOC geographical distribution and accessibility, analytical approach utilised (None-GIS or GIS approach. If GIS was used, for what purpose?), key findings reported, and interpretation of geographical findings. Data synthesis involved consolidation of data extracted from the retrieved articles. A mix of summary tables and charts was used to present the aggregated data to show trends and patterns for distribution and characteristics of the included studies. As study findings were varied, narrative synthesis was used to analyse and interpret the findings [13,14].

### Quality assessment

Building on a previous quality assessment framework proposed for EmOC indicators more broadly [15] and further guidance from the UN EmOC monitoring handbook [5], we developed a bespoke quality assessment framework for studies assessing EmOC geographical distribution and accessibility (Appendix S2 in **Online Supplementary Document**). A bespoke framework was required, as compared with the other 7 EmOC indicators, EmOC indicator 2, which focuses on EmOC geographical distribution and accessibility, requires unique methods for data collection and analysis [5]. This framework assessed the use of recent population figures, type of facilities included (public vs private), direct inspection for data collection, geo-referencing of EmOC facilities, information provided on methods used for assessment, mapping of EmOC facilities in relation to catchment population and identification of underserved areas. For scoring, 1 point was awarded for each achieved criterion and 0 point when not achieved or not particularly clear. Studies were assessed as high quality, if they achieved 75% or more of the criteria, medium quality for 50-74% or low quality for less than 50%.

## RESULTS

### Summary of results

**Figure 1:**
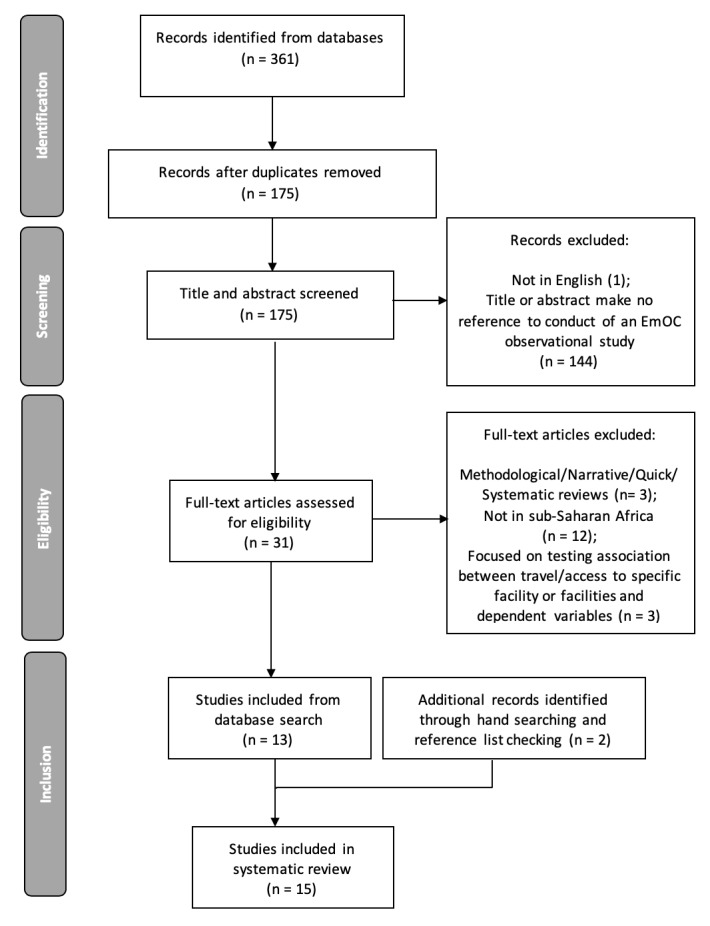
Flow diagram of the literature search process.

As shown in the PRISMA flow diagram, following removal of duplicates, we had 177 records from all databases combined. After abstract and full-text reading to specifically find articles that assessed EmOC geographical distribution and accessibility, 15 studies were found to meet the set inclusion criteria and were included for review (**Figure 1**).

### Quality assessment of studies

Of the 15 studies, seven were assessed to be of high quality [8,9,16-20], 4 were medium quality [21-24] and the remaining 4 studies were assessed as being of low quality [25-28] (See Quality assessment of studies in Appendix S2 in **Online Supplementary Document**).

### Distribution of included studies

**Figure 2:**
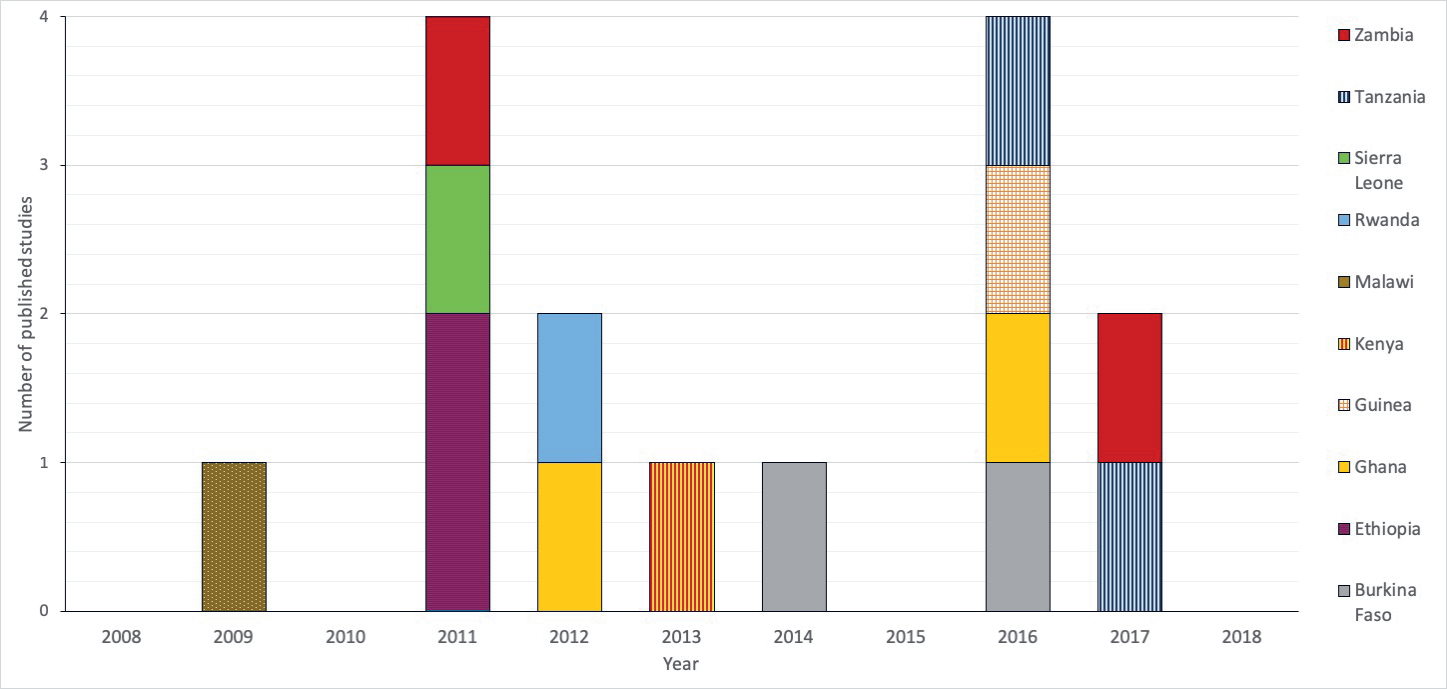
Distribution of emergency obstetric care (EmOC) geographical distribution published peer-reviewed studies in sub-Saharan Africa.

Since 2009, there has been an average of about 1 study per year that assessed EmOC geographical distribution and accessibility in SSA published in the peer-reviewed literature (**Figure 2**). The peak publication year was in 2011 [8,22,24,25] and 2016 [16,17,21,27] when 4 articles were published in each year (**Figure 2**). 2 studies have each been published in Burkina-Faso [26,27], Ethiopia [8,25], Ghana [17,19], Tanzania [20,21] and Zambia [22,28]. Guinea [16], Kenya [18], Malawi [23], Sierra-Leone [24], Rwanda [9] each have 1 published study (**Figure 2**).

### Characteristics of included studies

8 of the 15 studies were conducted on a national scale [16,17,19,22,24-27], while the remaining 7 studies assessed sub-national levels – regions or districts [8,9,18,20,21,23,28] (**Table 2**).

**Table 2:** Summary of study characteristics

Study characteristics	No. of studies (n = 15)	% of total
**Scale of study:**		
National	8	53.3%
Sub-national	7	46.7%
**Indicators assessed:**		
Indicator 1 & 2	8	53.3%
Indicator 1 & 2 plus another	1	6.7%
Five or more indicators	6	40.0%
**Assessment model**		
United Nations Emergency Obstetric Care assessment tool	12	80.0%
United Nations Emergency Obstetric Care assessment tool + Geographic Information Systems	3	20.0%
**Level of care assessed:**		
Basic and Comprehensive Emergency Obstetric Care	14	93.3%
Comprehensive Emergency Obstetric Care only	1	6.7%
**Type of facilities assessed:**		
Public facilities only	1	6.7%
Public and private facilities only	3	20.0%
Public, private and mission facilities	9	60.0%
Could not tell type of facility classification	2	13.3%
**Data sources used indicator assessment:**		
Secondary population and primary facility data	4	26.7%
Primary facility and geographical data	1	6.7%
Secondary population, facility and geographical data	4	26.7%
Secondary population, primary facility and primary geographical data	6	40.0%
**Geographical analysis and visualisation presented:**		
Yes	9	60.0%
No	6	40.0%
**Use of Geographic Information Systems (GIS, n = 9):**		
Thematic mapping	3	33.3%
Thematic mapping and spatial analysis	3	33.3%
Thematic mapping and spatial modelling	3	33.3%
**Interpretation and implication of findings presented:**		
Yes	8	53.3%
No	7	46.7%

8 articles focused on the UN EmOC indicator 1 and 2 (availability and geographical distribution) [8,9,17,19,20,22,26,28]. 1 study looked at both indicator 1 and 2 as well as indicator 5 [18], while the remaining 6 studies assessed 5 or more of the 8 EmOC indicators [16,21,23-25,27]. 12 studies used only the UN EmOC assessment guidelines as the basis for their analysis [8,16-18,21-28], while 3 studies used additional GIS frameworks not stipulated in the UN EmOC assessment handbook [8,19,20] (**Table 2**).

14 studies assessed both basic and comprehensive EmOC facilities [8,9,16-25,27,28], with 1 study focusing on only CEmOC facilities [26]. 9 studies assessed all types of facilities within the country or district (public, private and mission owned) [8,18,19,21-25,27]. 3 studies assessed public and private facilities [16,17,28] and 1 study assessed only public facilities [26]. EmOC classification of facilities could not be determined for 2 studies [9,20] (**Table 2**). 5 studies selected all possible facilities within their set geographical assessment area [9,16,18,19,23], while 3 studies selected all hospitals and randomly selected lower facilities like health centres [21,22,24]. Of the remaining studies, 4 sampled a proportion of the available facilities, ranging from 31% to 98.6% [8,17,25,27]. Proportion of hospitals sampled was not stated or could not be determined for 2 studies [20,28] (See data extraction sheet in Appendix S3 in **Online supplementary document**).

6 of the 15 studies used a combination of secondary population, primary facility and primary geographical data [8,9,22-25]. 4 used only secondary population and primary facility data [16,21,27,28] and another four studies used secondary population, facility and geographical data [17,19,20,22]. 1 study used primary facility and geographical data [26] (**Table 2**).

9 studies presented some information on their analytical framework for assessing EmOC geographical distribution and provided some visualisation in the form of a map [8,9,16-20,22,24]. The other 6 studies did not provide any detail and/or did not include a map [21,23,25-28] (**Table 2**).

In 6 studies, the authors simply counted number of facilities per district and estimated the locations of facilities on a map [21,23,25-28]. In 9 studies, the authors used a GIS software to aid analysis [8,9,16-20,22,24] (**Table 2**). For studies that used GIS, it was used for thematic mapping as either solely mapping locations of fully functioning facilities within districts in 1 study or to map fully functioning facilities and relate the output to the 500 000 population benchmark set by the UN [16,17,24]. In addition to mapping facilities, 3 studies used GIS for some form of spatial analysis, either to estimate straight-line distances between the facilities and place of residence of women while building concentric travel buffers (circles with a common centre) around the facilities [18,22] or relate spatial location of facilities to rate of EmOC service utilisation [9]. 3 studies used GIS for spatial modelling of various travel scenarios for women in need of EmOC within specified geographical areas [8,19,20] (**Table 2**). 8 of the 15 studies interpreted their findings within the discussion section and provided implication of their findings [8,9,18-20,22-24], while the remaining 7 studies did not provide any detailed interpretation of findings [16,17,21,25-28] (**Table 2**).

### Findings reported

Most studies reported inequitable distribution of CEmOC and BEmOC facilities. The sub-national studies mainly reported that there was concentration of CEmOC facilities in urban areas [21-23] (**Table 3**). The national survey conducted in Zambia pointed that more than 75% of those who reside in rural areas lived more than 15 km of an EmOC facility [22] (**Table 3**). In the Ghana national survey, across board, 34% and 50% of women lived more than 2 hours away from the nearest partial or fully functional EmOC and specifically CEmOC facilities respectively [19]. Similarly, 32% of live-births occurred in places where it was impossible for women to reach with motorised means of transport within 2 hours [20]. In the most rural areas, the figures rose to 63% and 81% [19] (**Table 3**). Within rural areas of Rwanda, CEmOC rates were the lowest in the more remote parts and incidence or morbidities and mortalities associated with pregnancy complications was higher than in less remote rural parts [9] (**Table 3**). Other surveys highlighted that there were more fully functioning EmOC facilities in central areas of the country or district [18,25] or in the capital [16,21,24] (**Table 3**). In some countries, even when there is “good geographical distribution of hospitals”, very few are fully functional [26] (**Table 3**).

**Table 3:** Summary table of key geographical findings

Author(s)	Year	Country of study	Indicators assessed	Scale of study	Number of facilities studied	Percentage of total facilities surveyed	Type of facility studied	Key geographical findings presented
Kongnyuy et al [23]	2009	Malawi	Indicator 1-6	Sub-national	73	100% of all facilities within the selected geographical area.	Public, private and mission facilities	There was no equitable distribution as some rural areas are not covered. Most of the Comprehensive EmOC* facilities were located in the central area of Lilongwe District and three were actually in or near the capital city.
Admasu, Haile-Mariam & Bailey [25]	2011	Ethiopia	All indicators	National	795	98.6% of all facilities within the selected geographical area.	Public, private and mission facilities	In Ethiopia, facilities were concentrated in the centre of the country, leaving peripheral areas underserved. Only 1 (Harari) of 11 regions met the goal of 5 per 500 000. The most populous regions of Oromiya, Amhara, and Southern Nations, Nationalities, and Peoples’ Region had only 0.4, 0.4, and 0.5 EmOC facilities per 500 000, respectively.
Bailey et al [8]	2011	Ethiopia	Indicator 1 & 2	Sub-national	249	31% of all facilities within the selected geographical area.	Public, private and mission facilities	Approximately 70% of the population of Tigray and Amhara regions is served by facilities that are within a 2-h transfer time to a hospital with obstetric surgery. By adding vehicles and communication capability, this percentage increased to 83%. In a second scenario, upgrading 7 strategically located facilities changed the configuration of the referral networks, and the percentage increased to 80%. By combining the two strategies, 90% of the population would be served by midlevel facilities within 2 h of obstetric surgery. The mean travel time from midlevel facilities to surgical facilities would be reduced from 121 to 64 min in the scenario combining the 2 interventions.
Gabrysch et al [22]	2011	Zambia	Indicator 1 & 2	National	1370	100% public hospitals. Percentage of private hospitals surveyed not reported.	Public, private and mission facilities	Geographic access to EmOC services in rural areas was low; in most provinces, less than 25% of the population lived within 15 km of an EmOC facility
Oyerinde et al [24]	2011	Sierra Leone	Indicator 1-6	National	145	100% of all hospitals. 33% of community health clinics and four MCH posts per district.	Public, private and mission facilities	Eastern Province and Southern Province had the lowest coverage. There was an abundance of Comprehensive EmOC facilities in Western Area District (where the capital city, Freetown, is located).
Gething et al [19]	2012	Ghana	Indicator 1 & 2	National	1864	100% of all facilities within the selected geographical area.	Public, private and mission facilities	A third of women (34%) in Ghana live beyond the clinically significant two-hour threshold from facilities likely to offer emergency obstetric and neonatal care (EmONC) classed at the ‘partial’ standard or better. Nearly half (45%) live that distance or further from ‘comprehensive’ EmONC facilities. In the most remote regions these figures rose to 63% and 81%, respectively.
Sudhof et l. [9]	2012	Rwanda	Indicator 1 & 2	Sub-national	9	100% of all facilities within the selected geographical area.	Could not tell	The lowest Caesarean section rates were found in the more remote part of the hospital catchment area.
Echoka et al [18]	2013	Kenya	Indicator 1, 2 & 5	Sub-national	40	100% of all facilities within the selected geographical area.	Public, private and mission facilities	All the three hospitals offering Comprehensive EmOC services and one of the two health centres offering BEmOC services were located in Malindi Division, the main urban centre in the district. The area is served by a relatively well functioning public transport system and adequate roads. The two vast and remote divisions, Langobaya and Marafa, were not served by any EmOC facility and are not connected to any major trunk road with regular public transport. Average distance to the nearest EmOC facility was 5kms and 30kms in the urban and rural areas respectively.
Compaoré et al [26]	2014	Burkina Faso	Indicator 1 & 2	National	52	100% of public hospitals. No private hospitals included.	Public hospitals only	Map of georeferenced facilities shows a relatively good geographical distribution of both regional and district hospitals within the country, very few of which are ready to provide Comprehensive EmOC.
Bosomprah et al [17]	2016	Ghana	Indicator 1 & 2	National	1159	91% of all facilities within the selected geographical area.	Public and private facilities	Greater Accra and Ashanti recorded a shortfall of 28 and 26 facilities, respectively, whereas Upper East and Upper West had a shortfall of only 7 and 3, respectively. Subnational analyses based on estimated total pregnancies in each district revealed a pattern of inequity in service provision across the country.
Baguiya et al [16]	2016	Guinea	All indicators	National	502	100% of all facilities within the selected geographical area.	Public and private facilities	There was a scarce and unequal distribution of such facilities. Fully functioning facilities were not equally distributed across regions. Boké and Conakry had four each, whereas Kindia and Mamou had none.
Kouanda et al [27]	2016	Burkina Faso	All indicators	National	1628 (2010) and 812 (2014)	82% (2010) of facilities at national level. Not estimated in the 2014 survey.	Public, private and mission facilities	There was wide regional disparity in both 2010 and 2014 on the availability of functional EmONC health facilities.
Fakih et al [21]	2016	Tanzania	Indicator 1-7	Sub-national	79	100% of all hospitals. 38% of Primary Health Care Units (PHCUs) across all districts.	Public and private facilities	The distribution of Basic EmOC and Comprehensive EmOC facilities varied across Zanzibar. Basic EmOC facilities were available in North Pemba and South Pemba regions, as well as West Urban regions. Comprehensive EmOC facilities were mainly concentrated in Urban West (Unguja); North Pemba and South Pemba regions.
Tembo et al [28]	2017	Zambia	Indicator 1 & 2	Sub-national	35	Could not tell	Public and private facilities	18 Basic EmOC per 500 000 population; 5 Comprehensive EmOC per 500 000 population.
Chen et al [20]	2017	Tanzania	Indicator 1 & 2	Sub-national	127	Could not tell	Could not tell	Of all live births in Kigoma Region, 13% occurred in areas where women can reach EmOC facilities within 2 h on foot, 33% in areas that can be reached within 2 h only by motorized vehicles, and 32% where it is impossible to reach EmOC facilities within 2 h. Over 50% of births in 3 of the 8 administrative councils had poor estimated access. In half the councils, births with poor access could be reduced to no higher than 12% if all female residents had access to motorized vehicles.

EmOC – emergency obstetric care

## DISCUSSION

Our findings showed that there are only a few studies assessing EmOC geographical distribution and accessibility in SSA published in the peer-review literature (15) [8,9,16-28]. Of the 46 sub-Saharan African countries [12], only 10 had peer-reviewed assessments. This is despite the huge burden of maternal deaths that can be addressed with improved EmOC access in the sub-region [2]. However, the finding of limited quantity of peer-reviewed studies is not surprising. A 2016 systematic review of peer-review literature showed that EmOC indicator 2 was 1 of the least studied EmOC indicator in LMICs [15].

In terms of quality of studies, evidence from our review suggests that studies that focused on indicator 1 and 2 only were of the highest quality [8,9,17-20]. These studies included more detail and better interpreted their findings. 4 of 6 studies judged to be of the highest quality were sub-national [8,9,18,20], and the other 2 were national studies [17,19]. A national and a sub-national study conducted by the same group of authors were assessed as low and high quality respectively [8,25]. Similarly, national studies conducted in Burkina-Faso and Guinea involving authors from the same institution were assessed as low and high quality respectively [16,27]. As such, it is difficult to conclude that study scope (national vs sub-national) influences quality of the study. The most prevalent reason for low-quality ratings in studies were authors not providing any detail of how they geo-referenced EmOC facilities and identified the catchment population for each assessed facility [9,21,23-28] and not mapping facilities in relation to population of the assessed district [23,25-28]. These criteria are recommendations in the UN assessment guidelines [5]. In our review, only about half of the studies presented details of analysis for assessing EmOC geographical distribution, provided a map [8,9,16-18,20,22,24] and interpreted their findings [8,9,18,20,22-24]. For the remaining studies [21,23-28], assessment of indicator 2 was essentially presented as an “add-on”, without going into any significant detail on assessment process or interpretation of findings. It was particularly surprising that no map was included in these studies, bearing in mind the strength of visualisation in strengthening an abstract indicator such as indicator 2 and the power of such tool for advocacy and planning [8].

A study included only public facilities [26], and in this study, this was the stated objective. No reason was given for the non-inclusion of private facilities. However, for some studies that included public and private facilities [16,17,28], it was not clear if inclusion of private facilities solely referred to “privately-owned” by an individual or if “mission-owned” facilities were also classified as private facilities. The WHO assessment guideline clearly identifies five categories of operating agencies – Government, private, nongovernmental organization, religious mission, and others [5]. In cases in which these operating agencies have not been included in the study, it is important that such non-inclusion is clearly stated within studies, as there might be implications for interpretation and comparison with similar studies. A statement showing the percentage distribution of EmOC facility types (public/private/non-governmental/faith-based), as was done by Baguiya et al [16], will make such distinctions clearer.

Only half of the reviewed studies used GIS for analyses [8,16-20,22,24]. Not just for EmOC assessments, but generally within maternal and newborn health (MNH), mapping and application of GIS has been lagging behind, despite its more robust and sophisticated application in many other health-related fields where it has proven to be a valuable tool for generating evidence to aid strategic decision-making [29]. A 2015 review found only 19 GIS applications in MNH, published in Africa [30]. In our review, we found that GIS application was mainly limited to simply mapping EmOC facilities or relating EmOC facility numbers to the 500 000-benchmark population recommended by WHO. However, focus on this benchmark only reflects EmOC service ‘provision’ at sub-national levels. While ‘provision’ is important, there is a critical need to demonstrate ‘access’ to and ‘utilisation’ of those facilities by women, which is only possible if more is done with GIS [8,30]. A more useful finding for EmOC service planners would be EmOC coverage, which can show for example that there are 2 comprehensive EmOC facilities available for a million population, and all women in the district can reach a facility within two hours, irrespective of their means of transport [20,22]. Only a third of the studies in our review provided such sophisticated yet critical level of analyses [8,18-20,22]. Clearly, there is scope for leveraging more of the potentials of GIS in producing for useful results for service planners.

For those who used GIS to assess travel to facilities, some estimated straight-line distances between facilities and residences of women [18,22]. While this may partly reflect access, estimating straight-line (Euclidean) distances do not demonstrate real-life travel experiences of women to EmOC facilities, since the route of travel may be convoluted, with poor conditions and different terrain barriers [18]. Therefore, interpretation of straight-line buffers can be complicated. Some women may fall within the buffer but may not be able to access facilities because geographical bodies such as mountains and rivers are located between their residence and the facilities. However, the accuracy derived from real-life travel estimates should always be juxtaposed with the associated cost and complexity of estimating Euclidean distances. In a study in Ghana, some authors showed that Euclidean distances can be reasonable proxies for the actual distance covered in LMICs [31]. More research is required in other LMICs to ascertain this finding. However, in the few studies that reported access and coverage, it appeared that generally about a third of women lived more than two hours away from functional EmOC facilities [19,20]. This is similar to conclusions made in a recent analysis of access to emergency hospital care provided by the public sector in SSA, in which the authors showed that 28.2% of women of reproductive age needed more than two hours to reach the nearest hospital [32]. When disaggregated, wider disparity to accessing EmOC was reported in rural areas in SSA, with rural women twice more likely to live more than two hours away from functioning EmOC facilities than urban women [19].

In our review, studies that used the UN EmOC assessment framework along with more elaborate GIS assessment framework appeared to provide more detailed analysis and interpretation of their findings [8,19,20]. There is a need to rethink the scope of the UN assessment framework for Indicator 2, so that more critical information, which had previously been labelled “supplementary” and “cumbersome to analyse” [5], can be generated. In other areas of health in which GIS has been applied without a specific framework for supporting assessments, authors have been able to detail more extensive analysis with useful information for service planners [33-36]. Additional data such as health worker density and hours of service should be considered for future assessments. This adds crucial information on functionality of facilities for service planners and indeed will highlight the fully functional EmOC facilities, which are expected to be open 24 hours a day, seven days a week [22]. Such complementary data can be collected using tools such as the Service Availability and Readiness Assessment (SARA) survey [37] and the Workload Indicators of Staffing Need (WISN) survey [38].

GIS clearly provides further insight for EmOC geographical distribution and accessibility. So, why is it not used in all studies? 1 study in our review stated this was because of the “non-availability of GIS software to analyse geographical data” [28]. It is not clear why this was the case for this study, more so in the post-2010 era of the GIS evolution, when GIS is described as being ubiquitous, even in SSA [39]. In a previous review, issues such as prohibitive cost of GIS assessments and need for advanced technical know-how have been attributed to its limited use in EmOC assessments [15]. However, recent mobile technological developments, availability of free population data and in some cases facility data bring new opportunities for scaling up GIS use for EmOC assessments in SSA [29].

Previously, data collection for EmOC indicator 2 assessment required use of handheld global positioning system (GPS) machines, which came at a cost to the researchers and required knowledge on how to import the geo-coordinates and post-process into specialized software after collection [15]. However, with the proliferation of smart mobile phones in many LMICs [40], free applications such as ‘Get Geo-Coordinates’ (Available in Android Play Store) and ‘Easy GPS’ (Available in Apple App Store) can be used to capture location data for assessments, without incurring significant costs aside from travel-related expenses. For researchers who are unable to travel to the EmOC facilities and/or residences of women, there are free websites, such as http://www.gps-coordinates.net/ and http://www.mygeoposition.com/ that can be assessed from office-based desktops, which provide x,y geo-coordinates for specific addresses/locations. No advanced technical know-how is required to use these applications or websites. However, ethical considerations regarding geo-referencing specific locations require researchers to randomly displace the coordinates to guarantee confidentiality. Similar random displacements are carried out in the conduct of the Demographic Health Surveys conducted in LMICs [41].

As with GIS for data collection, the basic analysis for geographic distribution of EmOC facilities can be easily done today. It involves simply geocoding the x,y coordinates that have been collected in data files known as shapefiles (which include a feature geometry file (.shp), positional index (.shx) and some attribute data (.dbf) [42]). Though availability of these shapefiles may be limited for some LMICs [43], there are growing databases online such as http://www.diva-gis.org/Data and http://www.gadm.org/, from which shapefiles at national and sub-national levels can be downloaded for free. The supplementary analyses suggested in extant literature can then be performed using these shapefiles with various attributes. Typically, these supplementary analyses need to be done within proprietary GIS software such as ArcGIS^®^ (Environmental Systems Research Institute, Redlands CA, USA) or MapInfo^®^ (Pitney Bowes, Stamford CT, USA). However, there are now free open-source alternatives such as GRASS GIS^®^ (GRASS Development Team, Bonn, Germany), QGIS^®^ (QGIS Development Team, Global) and AccessMod^®^ (enviroSPACE Laboratory, Geneva, Sitzerland) that are increasingly becoming more user-friendly and designed to be used by non-specialists. If these tools appear complex, Google Maps^®^ (Google, Mountain View CA, USA), a freely available tool used for everyday commuting, can be used to estimate travel time and distance. Google Maps^®^ also has the capability to provide data on live and typical traffic behaviour.

To the best of our knowledge, this is the first systematic review that specifically explores assessment of EmOC geographical distribution and accessibility in SSA. In interpreting our findings, some limitations need to be kept in mind. First, our search is limited to peer-reviewed literature. There are published and unpublished assessments in the grey literature, which we have not included. In addition, we have only included studies published in English language.

## CONCLUSION

Assessment of EmOC geographical distribution and accessibility is limited in SSA. With the advent of smarter mobile technology and explosion of innovative, user-friendly open-source GIS technologies, there is a unique opportunity for scaling-up quantity and quality of such assessments in the sub-region. Where skill gaps still exist, EmOC assessors and service planners should collaborate with GIS specialists. Furthermore, these assessments need to be able to provide answers to questions regarding service provision, access, coverage and identify priority areas for new or upscaled EmOC facilities [44]. These answers are critical components in the efforts to reduce maternal mortality in SSA.

## Additional material

Online Supplementary Document
